# Splenic Infarction Associated With Epstein-Barr Virus in an Adult With an Anatomic Anomaly: A Case Report

**DOI:** 10.7759/cureus.40530

**Published:** 2023-06-16

**Authors:** Kazuki Kobayashi, Yoji Kishi, Yusuke Serizawa, Yoshifumi Kimizuka, Hideki Ueno

**Affiliations:** 1 Surgery, National Defence Medical College, Tokorozawa, JPN; 2 Internal Medicine, National Defence Medical College, Tokorozawa, JPN

**Keywords:** anatomical anomaly, infectious mononucleosis, epstein-barr virus, splenomegaly, splenic infarction

## Abstract

Splenic infarction (SI) is often associated with circulatory and hematological diseases and infections. Here, we report a rare case of SI in an adult with infectious mononucleosis (IM) caused by the Epstein-Barr (EB) virus. A 31-year-old male with an unremarkable medical history presented with abdominal pain and fever. Contrast-enhanced computed tomography revealed focal SI. The splenic artery branching from the superior mesenteric artery was <5 mm in diameter. The diagnosis of EB virus infection was made based on physical examination and blood test results. As no evidence of cardiogenic disease, malignant lymphoma, or other infections were present, a diagnosis of SI associated with IM was made. A symptomatic treatment was administered, and the splenomegaly and SI improved two weeks after discharge. IM was assumed as the cause of the focal SI.

## Introduction

Splenic infarction (SI) can be caused by cardiogenic emboli, atrial fibrillation, infective endocarditis, lymphoma, collagen disease, or infections. SI is often asymptomatic, rarely results in splenic rupture or hemorrhage [1], and requires careful follow-up when diagnosed.

Infectious mononucleosis (IM) is an infectious disease caused by the Epstein-Barr (EB) virus [2], and approximately 90% of cases occur in early adulthood [3]. IM in childhood is mostly asymptomatic, but young adults frequently present with symptoms including fever, pharyngitis, and cervical lymphadenopathy [4]. Blood tests reveal a significant increase in peripheral blood lymphocytes [4].

Cases of SI associated with IM have rarely been reported, and the true incidence is unknown [5]. Here, we describe a case of a 31-year-old man with SI associated with IM.

## Case presentation

**Figure 1:**
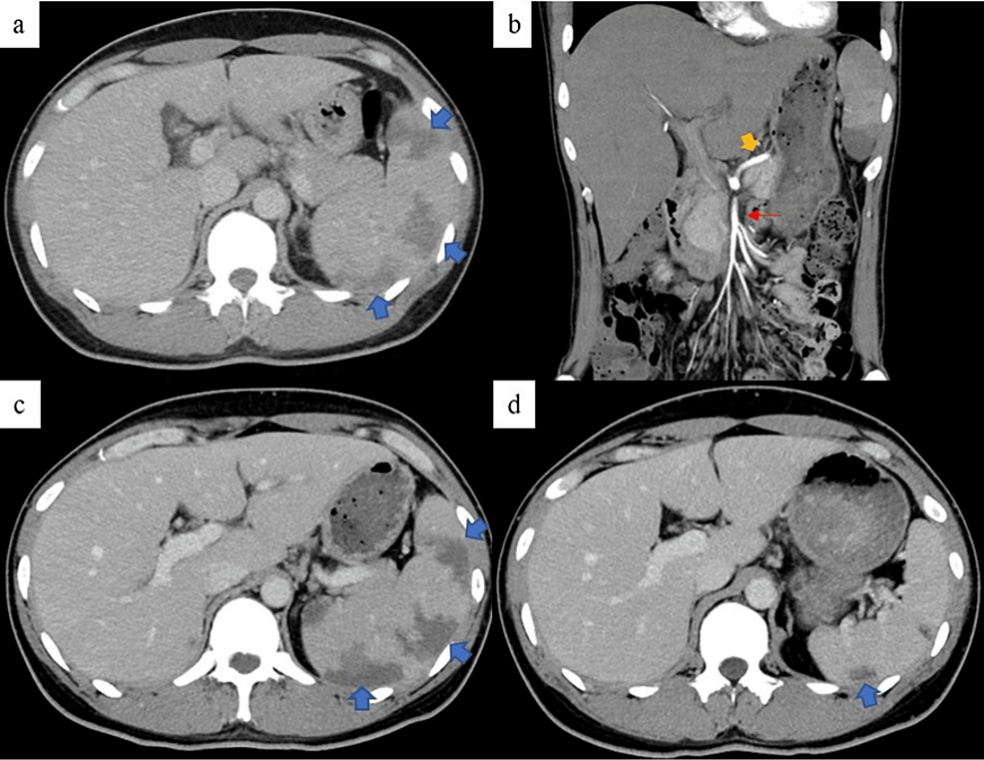
Abdominopelvic computed tomography image with the splenic infarction indicated by the blue arrows. (a) is an axial image at admission. Splenomegaly with localized splenic infarction can be observed. The splenic volume was 526 mL. (b) is a coronal image at admission. The splenic artery (SpA; orange arrow) branches from the superior mesenteric artery (red arrow). The maximum diameter of the SpA was 4.9 mm. (c) is an axial image obtained on the fourth day of hospitalization. Splenomegaly remains unchanged, and there is no enlargement of the infarcted area. (d) is an axial image obtained two weeks after discharge. Splenomegaly has improved markedly, and the infarcted area has shrunk. The splenic volume was 228 mL.

A 31-year-old man with no remarkable medical history presented with high fever and right hypochondriac pain. A nearby physician prescribed antipyretic medication and monitored his progress; however, his high fever and abdominal pain persisted for more than one week. The patient was transferred to a local hospital. Abdominal contrast computed tomography (CT) revealed splenomegaly (splenic volume: 526 mL) and SI (Figure 1a). The splenic artery (SpA) and common hepatic artery (CHA) branched from the superior mesenteric artery (SMA), but there was no evidence of SpA obstruction (Figure 1b). The patient was then transferred to our hospital for further examination and treatment.

The physical examinations revealed pharyngalgia and striking pain in the right hypochondrium. Blood tests revealed a white blood cell count of 11300/μL, with 11.6% of neutrophils and 70.9% of lymphocytes, an aspartate aminotransferase (AST) level of 183 IU/L, an alanine aminotransferase (ALT) level of 245 IU/mL, a lactate dehydrogenase (LDH) level of 853 IU/L, and a C-reactive protein level of 0.7 mg/dL. The results of the coagulation tests showed a slightly elevated D-dimer level (4.7 μg/mL) and decreased activities of protein C (63%) and protein S (63%). Tests for antinuclear antibodies, lupus anticoagulants, anti-cardiolipin antibody Immunoglobulin G (IgG), and rheumatoid factor (RF) were negative. Serological tests revealed that IgG and immunoglobulin M (IgM) for EB viral capsid antigens were negative and positive, respectively. IgG for EB virus nuclear antigen (EBNA) was negative. Serological tests for cytomegalovirus, hepatitis B and C, and human immunodeficiency virus (HIV) yielded negative results. There were no electrocardiographic abnormalities; the echocardiographic findings were not suggestive of valvular disease or infectious endocarditis.

The patient was diagnosed with SI associated with IM secondary to acute EB virus infection. We treated the patient symptomatically, and his fever resolved, but his right quadrant tenderness persisted. Therefore, a contrast CT scan was performed on the fourth day of hospitalization. The SI did not worsen (Figure 1c), and blood tests showed a decrease in AST, ALT, and LDH levels. Therefore, the patient was discharged on the fifth day of hospitalization. Physical examination two weeks later at the outpatient clinic showed a complete remission of the right upper quadrant tenderness, and liver function tests and LDH levels returned to normal ranges. The splenomegaly improved (splenic volume: 228 mL; 43% volume reduction), and the SI area decreased (Figure 1d).

## Discussion

We report a rare case of SI associated with IM due to acute EB virus infection. In general, the causes of SI are as follows: 1) blood or bone marrow malignancy; 2) hypercoagulation states, including protein C or S deficiency; 3) thromboembolic disorders, including atrial fibrillation; 4) infection, including infective endocarditis; and 5) autoimmune disorders [6,7]. The frequency of SI due to IM remains unknown. Im et al. [1] reported that among 353 cases of infection-associated SI, only one (0.3%) was caused by the EB virus.

The mechanism of SI caused by IM is not clear, but several factors have been considered. First, the splenomegaly associated with EB virus infection may result in blood flow disturbances [5]. However, relative ischemia alone did not appear to be the cause of partial SI in the present case because the partial spleen was not enhanced even in the equilibrium phase of contrast CT. Second, microthrombus formation may have been triggered by the EB virus infection because of an underlying hematologic disease, such as hereditary spherocytosis [8], protein C activity deficiency [9], or sickle cell disease [10]. Third, EB virus infection may result in hypercytokinemia. Tumor necrosis factor (TNF)-α, a cytokine, is thought to inhibit the lysis of intravascular fibrin and form microthrombi by promoting the production of plasminogen activator inhibitor type-1, a fibrinolysis inhibitor [11].

There are two distinctive features of the case we reported. First, SI associated with the EB virus infection was reported for the first time in a young adult. The patient tested negative for EBNA IgG and positive for EB virus-viral capsid antigen IgM, suggesting a recent infection. It has been suggested that adolescents infected with the EB virus for the first time recruit large numbers of previously produced cross-reactive memory T cells in response to other viral infections, and IM occurs as a result of overreaction by the immune system [12]. In this case, we speculate that the excessive immune response caused a cytokine storm, which resulted in the suppression of protein C and S production and led to partial SI. Protein C and S production was considered a secondary effect. Second, there were anatomical variations in the SpA. Anatomical variants in which the SpA diverges from the SMA have been reported in less than 1% [13]. In a previous report, anatomical variations in the vascular anatomy were cited as a cause of SI [14]. In another study, the average SpA diameter was 5.9 mm on average [15]. In this case, the maximum SpA diameter observed was 4.9 mm. In addition, the maximum SpA diameter at the time of symptom remission was consistent at 4.7 mm.

There is no previous literature that mentions the diameter of the SpA branching from the SMA, but the smaller diameter of SpA in the present case might have been a factor related to the microembolization. Furthermore, focusing on the diameter of the splenic vein in this case, the maximum diameter was 12 mm at the time of admission, whereas it was 8.9 mm and thinner at the time of symptom remission. This suggested that the demand for blood in the spleen increased at the onset of IM, and the narrow-diameter SpA could not keep up with the supply, resulting in focal SI.

## Conclusions

SI associated with IM caused by EB virus in adults is rare. The cause of SI is unclear, but it has been suggested that an excessive immune response may be involved. In addition, a remarkable feature of this case was the anatomical variation of the SpA. The small vascular diameter may have caused a relative insufficiency of blood flow in the enlarged spleen due to IM. Further accumulation of cases is needed to determine the cause.
